# Temporal development of subdural collections in infants with confessed abusive head trauma: a forensic neuroimaging study

**DOI:** 10.1007/s00330-024-11144-1

**Published:** 2024-10-28

**Authors:** Maria Hahnemann, Bernd Karger, Alexander Radbruch, Hans-Joachim Mentzel, Daniel Wittschieber

**Affiliations:** 1 Department of Diagnostic and Interventional Neuroradiology and Pediatric Neuroradiology, University Hospital Bonn, University of Bonn, Bonn, Germany; 2 Institute of Legal Medicine, University Hospital Münster, University of Münster, Münster, Germany; 3 Section of Pediatric Radiology, Institute of Diagnostic and Interventional Radiology, Jena University Hospital, University of Jena, Jena, Germany; 4 Institute of Legal Medicine, University Hospital Bonn, University of Bonn, Bonn, Germany

**Keywords:** Child physical abuse, Forensic radiology, Estimating the time of injury, Tadpole sign

## Abstract

**Objectives:**

Estimating the age of injury in pediatric abusive head trauma (AHT) is a challenging task but potentially valuable for the identification of perpetrators. The aims of the study are (1) to describe the temporal development of different imaging features of subdural collections (SDCs), and (2) to provide novel age-diagnostic reference data for forensic-radiological expert reports.

**Methods:**

Using a multi-center approach and a 10-year study period, serial neuroimaging studies of 13 comprehensively investigated AHT cases (8 CT and 26 MRI scans) were analyzed regarding several subdural imaging parameters (SDC appearances, entities, components, and associated findings). Due to confessions by perpetrators, the time points of the trauma were presumed unique and known in all cases facilitating correlation of imaging findings with time.

**Results:**

Hyperdense SDCs in CT were found up to 9 d post-injury (p.i.), CSF-like SDCs in CT or MRI as early as from the 3rd hour p.i., and subdural membrane formation as late as from day 283. The heterogeneous variant of the subdural hematohygroma was observed to be the dominant SDC entity between 3 h and 22 d p.i. The tadpole sign was detected in MRI between 3 h and 46 d p.i.

**Conclusion:**

Certain subdural imaging findings may be helpful for estimating the age of injury in AHT. Subdural membrane formation is demonstrated to be a late finding and the tadpole sign is an early phenomenon p.i. The data corroborated that the sediment but not the supernatant has the potential for being valuable for age-diagnostic aspects.

**Key Points:**

***Question*** *Reliable evidence-based data on the development of SDCs is sparse but required for expert opinions on pediatric AHT*.

***Findings*** *Reference data on the evolution of the imaging appearance of SDCs and associated phenomena in confirmed cases of AHT are provided*.

***Clinical relevance*** *As there is a great need for estimating the age of injury in criminal and civil proceedings, many clinical radiologists are confronted with the diagnostic and forensic aspects of AHT that are addressed in the present study*.

## Introduction

Abusive head trauma (AHT) describes a potentially fatal head injury of children due to either forceful shaking (shaken baby syndrome, SBS), blunt force trauma, or a combination of both [1–3]. With about 20–30 cases in 100,000 live births, AHT is a relatively common phenomenon and the predominant cause of morbidity and mortality in children younger than 2 years with traumatic brain injury [4–6].

AHT is characterized by the presence of typical, but neither obligatory nor evidentiary, findings such as subdural collections (SDCs), retinal hemorrhages, or acute encephalopathy. The subdural hematoma (SDH) is classically referred to as the most common SDC in AHT [7]. Additionally, other SDC entities occur, even in the early phase. Recently, the heterogeneous variant of the subdural hematohygroma (SDHHy hete) was observed to be the most common SDC entity in initial neuroimaging of AHT cases reflecting the significant role of possible early post-traumatic cerebrospinal fluid (CSF) influx [8].

Thus, the SDHHy hete may play a key role in AHT case assessment. It can be characterized as an SDC formed by two components that coexist within the same subdural compartment. These components can usually be distinguished from one another (fluid–fluid levels) and may appear hyper- and hypodense compared to brain parenchyma in initial CT scans. The hypodense part can be interpreted as either acute subdural hygroma (SDHy; CSF/CSF-like collection, e.g., due to an arachnoid tear), or supernatant of a sedimented SDH (blood changed by gravity) [8, 9].

There is general consensus that precise dating of SDCs by means of neuroimaging, which is often requested by courts in criminal proceedings, is unrealistic [3, 7–10]. However, this does not mean that any time-related statements on SDCs are impossible. Serial neuroimaging data of SDCs and the correlation with clinical presentation, reported history, and additional neuroimaging findings can enable experts to at least estimate the date of injury, which can be relevant not only in forensics but also for clinical questions. Therefore, reliable evidence-based data on SDC development, on which forensic expert opinions can be based, is required. Such data are inherently sparse as the date of injury is only rarely known.

Hence, the present study analyzes serial neuroimaging studies of well-investigated AHT cases with corroborated dates of trauma. By means of CT and MRI, the temporal development of SDCs, SDC components, and SDC-associated phenomena is described, and novel reference data for age estimation practice (supplementing the small pool of previous data) is provided.

## Materials and methods

Our retrospective multicenter study at the three Institutes of Legal Medicine of the Universities of Cologne, Essen, and Münster between 2006 and 2015 originally comprised 72 AHT cases [8, 11–13]. The criteria used for diagnosing AHT have been described in detail previously [8, 13]. The resulting medico-legal expert opinions can be regarded as the best reference standard for AHT in Germany.

The original study cohort (*n* = 72) included 15 cases of AHT, at that time diagnosed as “shaken baby syndrome” (shaking alone) and confirmed by confession, which forms the basis of the present study. For all cases, complete clinical records including medical histories, demographic characteristics, and radiological imaging material were available (from birth up to the preparation of the expert opinion). Only pre-surgical imaging material was considered. In addition, our study group had special permission to analyze related court records, which contained all criminal investigation reports by the police, testimonies, expert opinions, and court judgments. In all 15 study cases, exact descriptions of the time points of AHT were found in confessions by perpetrators documented within the court records, in part additionally corroborated by other witnesses.

One board-certified radiologist and one board-certified forensic physician (both with more than 10 years of specific experience in neuroimaging of child abuse cases) reviewed all imaging studies in cooperation. All CT and MRI imaging studies were analyzed with regard to the presence of SDCs. In the rare case of non-concordant results, a board-certified senior radiologist with more than 20 years of specific experience arbitrated until consensus was achieved.

In CT, the following parameters were recorded:

- 1. Density categorization of the SDC as mixed, hyperdense, or hypodense compared to brain parenchyma.
- 2. Presence of subdural hyperdensities, in general.

In MRI, the following parameters were recorded:

- 1. Presence of subdural clot and/or presence of sedimented blood. The term “clot” was defined as circumscribed blood formation not yet changed by gravity. “Sedimented blood” was defined as descended blood components within an SDC.
- 2. Signal intensity of supernatant and sediment in different sequences (T1, T2, T2*/SWI, and FLAIR) categorized as hypointense, isointense, and hyperintense compared to brain parenchyma.

In CT and MRI, the following parameters were recorded:

- 1. Presence of tentorial sediment (in MRI with and without the usage of a T2*/SWI sequence).
- 2. Assignment of the “dominant SDC entity” according to Wittschieber et al [9] (Table 1):
  - SDH.
  - SDHy.
  - Subdural hematohygroma, homogeneous variant (SDHHy homo).
  - Subdural hematohygroma, heterogeneous variant (SDHHy hete).
  - Chronic subdural hematoma (cSDH).

**Table 1:** Definition of the five SDC entities according to Wittschieber et al (2019) [9]

Schematic drawings (CT appearance)	Definition
	SDH: According to established descriptions of the hyperacute, acute, and subacute stages of SDHs (summarized in [9]), SDH was defined as either homogeneous iso-/hyperdense SDC in CT, or homogeneous iso-/hyperintense SDC in MRI (T1), confirmed by corresponding signal intensities in T2 and FLAIR sequences.
	SDHy: Defined as homogeneous SDC, whose appearance in CT or MRI corresponded to CSF or CSF-like fluid (hypodense/CSF-isointense), without evidence for blood, blood products, or neomembranes.
	SDHHy homo: Defined as homogeneous SDC, whose appearance in CT or MRI did not correspond to pure CSF, but to a mixture of blood and CSF.
	SDHHy hete: Defined as SDC consisting of two opposite components (hypodense/-intense and hyperdense/-intense) that coexist within the same subdural location and may be clearly distinguished from one another, e.g., by fluid–fluid levels.
	cSDH: Defined as mostly heterogeneous SDC surrounded by and sometimes also subdivided into compartments by neomembranes and septa. The resulting subdural chamber formationsmay enclose fluid collections of different densities/signal intensities and may have multiple fluid–fluid levels.
- 3. Presence of subdural membranes.
- 4. Presence of the tadpole sign (round- to an oval-shaped blood clot in or next to an expanded bridging vein) according to Hahnemann et al [14] (in MRI with and without the usage of a T2*/SWI sequence).

### Statistical analysis

Continuous variables, such as the age of the child and time of imaging after trauma were shown as ranges, means, and medians. Categorical variables and qualitative parameters such as sex, type of trauma, type of imaging study performed, and characteristics of SDCs were reported as numbers.

## Results

### General characteristics of the study population

A total of 15 cases of confessed AHT with a known time of the trauma were recorded during a 10-year period. Two cases were excluded due to known repeated AHT at different time points. Thus, the final study cohort consisted of 13 cases (four females and nine males) aged between 1 and 5 months (mean 3 months and median 2 months). In all 13 cases, the confessed mechanism of AHT was specified as “violent shaking of the child”.

The medical records of all children did not reveal any birth-related trauma or other complications during birth. All children were born at term, except for one (born in the 34th week of pregnancy). No child showed noticeable abnormalities in body weight.

Within the time period from birth to the preparation of the medico-legal expert opinion, no child died.

All cases showed SDCs on the initial scan independent of the imaging modality used. Table 2 shows a synopsis of the imaging modalities and the imaging times performed in the 13 study cases. In total, 34 imaging studies could be analyzed: 8 CT scans (between 3 h and 23 d after the trauma) and 26 MRI scans (between 3 h and 490 d after the trauma).

**Table 2:** Synopsis of the imaging modalities and the imaging times performed in the study cohort

	Number of cases	Number of scans	Time after trauma		
			Range	Mean	Median
Imaging (total)	13	34	3 h–490 d	54 d	8 d
CT	7	8	3 h–23 d	5 d	23 h
MRI	11	26	3 h–490 d	69 d	11 d
Initial imaging (total)	13	13	3 h–46 d	5 d	1 d
CT	4	4	3 h–23 h	11 h	7 h
MRI	9	9	3 h–46 d	7 d	3 d
Follow-up studies (total)	8	21	1 d–490 d	308 d	21 d
CT after the initial CT scan	1	1	5 d	–	–
CT after the initial MRI scan	3	3	1 d–23 d	11 d	9 d
MRI after initial CT scan	2	2	1 d–3 d	2 d	–
MRI after initial MRI scan	7	15	2 d–490 d	102 d	23 d
● 1 follow-up	2	2	41 d–476 d	259	–
● 2 follow-ups	3	6	11 d–490 d	99 d	21
● 3 follow-ups	1	3	9 d–173 d	68 d	22
● 4 follow-ups	1	4	2 d–283 d	109 d	75 d

*h* hours, *d* days

### Imaging findings

The findings of CT and MRI regarding the evaluation of maximum and minimum times of the occurrence of different SDC characteristics are summarized in Table 3.

**Table 3:**
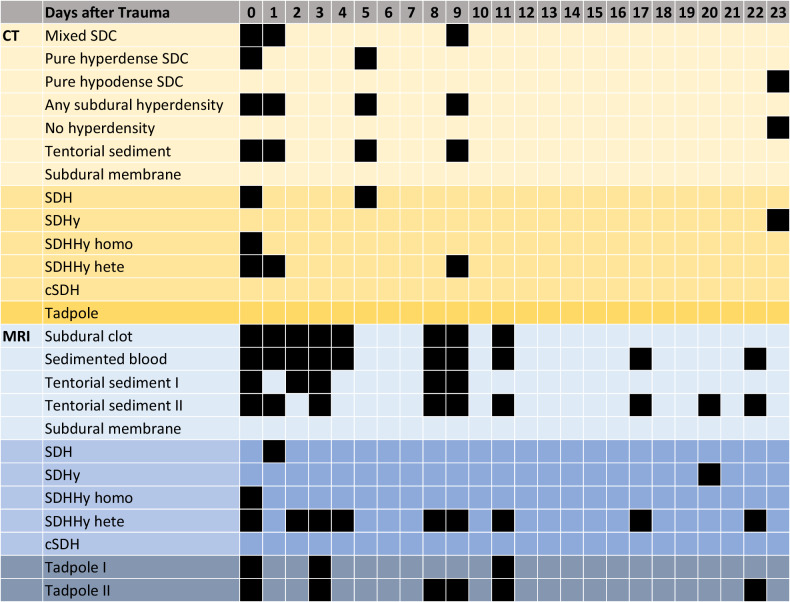
Illustrative summary of SDC characteristics observed in CT and MRI over time

The presence or absence of a respective finding is shown from day 0 to day 23 after trauma. Black squares indicate the presence of a respective finding. The finding “tentorial sediment” was differentiated between sediment detected without T2*/SWI (tentorial sediment I) and with T2*/SWI (tentorial sediment II). The finding “tadpole sign” was differentiated between the tadpole sign detected without T2*/SWI (Tadpole I) and with T2*/SWI (Tadpole II)

#### CT findings

**Fig. 1:**
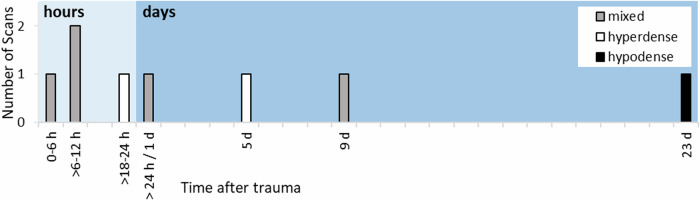
Densities of SDCs at different times after trauma observed in CT

CT scans showed primarily hyperdense and mixed SDCs (7/8 CT scans) between 3 h and 9 d after trauma. Only one hypodense SDC was found on day 23 after trauma (Fig. 1 and Table 3). Hyperdensities in general (7/8 scans), as well as tentorial sediment (6/8 scans) were present until day 9 after trauma (Table 3). No hyperdensities, in general, were found in the CT scan on day 23 after trauma, and no tentorial sediment was found in a CT scan beyond 9 d after trauma (Table 3).

**Fig. 2:**
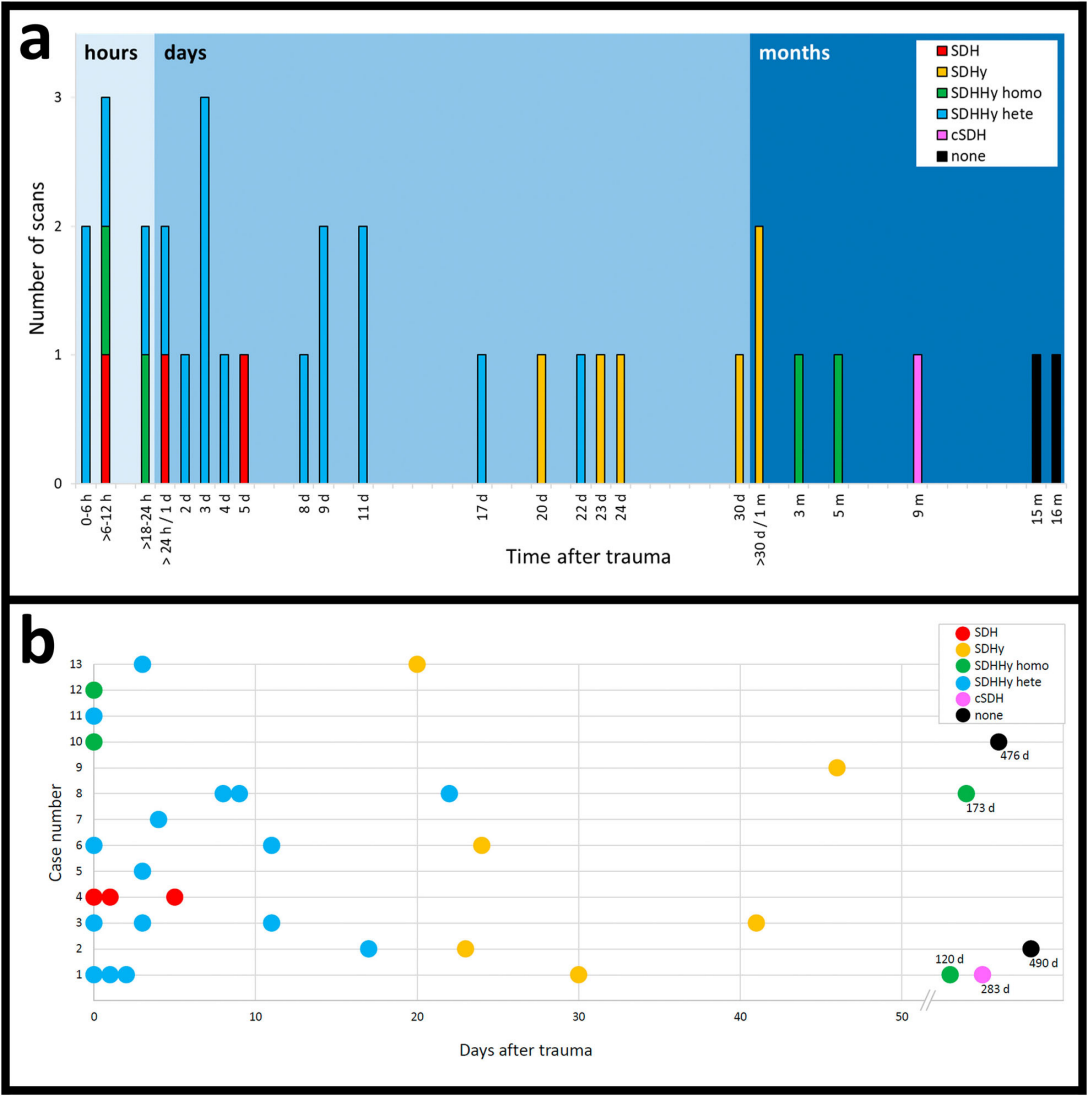
Dominant SDC entities observed in CT and MRI at different times after trauma. The number of scans does not add up to *n* *=* 34 because, firstly, in one case the CT and MRI scan were performed on the same day (and revealed the same result), and secondly, one MRI was not assessable regarding the dominant SDC entity due to incomplete data. **a** Presentation of the number of scans of all 13 cases. The timeline is divided into three different time sections (hours, days, and months). Note that, for example, SDHHy hete was found in 4 of 8 CT scans and in 13 of 16 MRI scans. **b** Presentation of each individual case (no. 1–13) on its own line. For reasons of clarity, the five scans after day 50 are shown on an artificially shortened timeline and with a directly assigned number of days after trauma

SDHHy hete (4/8 scans) was found to be the most common dominant SDC entity in the time period between 3 h and 9 d after trauma. One SDHHy homo was present 7 h after trauma. SDH (2/8 scans) was observed between 23 h and 9 d after trauma. One SDHy was found 23 d after trauma (Fig. 2).

None of the CT scans (0/8 scans) showed subdural membrane formation or a tadpole sign.

#### MRI findings

**Fig. 3:**
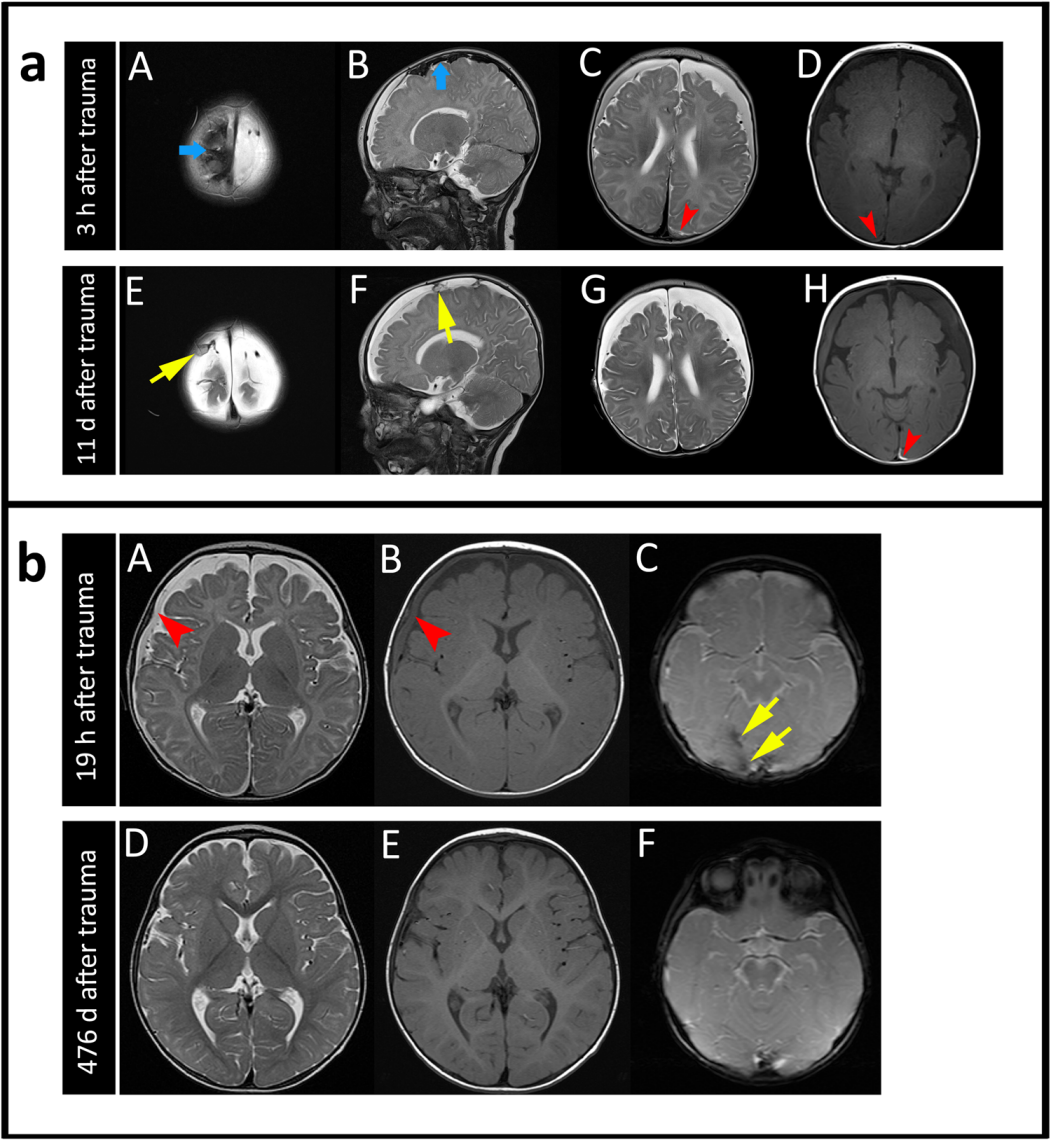
**a** MRI follow-up study of a 3-month-old boy showing a noticeable decrease of a subdural clot in a dominant SDHHy hete within 11 days. The figure shows axial T2w imaging proximal to the vertex (A *+* E), sagittal T2w imaging at the right hemisphere (B *+* F), axial T2w imaging (C *+* G), and axial T1w imaging (D *+* H). The detectable subdural blood clot (blue arrows, A *+* B) at the right parietal convexity 3 h after trauma decreased noticeably in a follow-up MRI scan 11 days after trauma. Instead, a tadpole sign at the right parietal convexity became visible (yellow arrows, E *+* F). Axial T2w imaging (C) and axial T1w imaging (D *+* H) showed an SDHHy hete with subdural sediment dorsally of the occipital lobes 3 h and 11 days after trauma (red arrowheads, C *+* D *+* H). **b** MRI scans of a 5-month-old girl at the time of the first scan showed complete regression of an SDHHy homo within 476 days. Initial MRI 19 h after trauma with axial T2w (A), T1w (B), and C T2*w imaging (C) showed a dominant SDHHy homo mainly at the right parietal convexity (red arrowheads), widened subdural spaces filled with fluid in both frontal regions, and small amounts of tentorial sediment in T2*w imaging (C) (yellow arrows). In a follow-up MRI 476 days after trauma no SDC was detectable in T2w (D), T1w (E), and T2*w imaging (F). Even widened subdural spaces and tentorial sediment in T2*w images (F) have disappeared

MRI revealed subdural clots between 3 h and 11 d after the trauma (10/26 scans), exemplified in Fig. 3a. Two MRI scans on day 3 and 1 MRI scan on day 11 after trauma did not show subdural clot formation (3/26 scans). No subdural clot was found in all MRI scans from day 17 after trauma (12/26 scans). One of the MRI scans on day 2 after trauma was not able to be evaluated for the presence of a clot due to incomplete data (1/26 scans).

Sedimented blood was present in 16 of the 26 MRI scans between 3 h and 22 d after trauma (16/26 scans) (Table 3). No sedimented blood was found in 1 MRI scan 20 d after trauma (1/26 scans) and in all MRI scans from day 24 after trauma (9/26 scans). Tentorial sediment was found in seven MRI scans without using T2*/SWI between 3 h and 9 d after trauma (Table 3). Furthermore, tentorial sediment was detected in 16 MRI scans using T2*/SWI between 3 h and 173 d after trauma, exemplified in Fig. 3b.

**Fig. 4:**
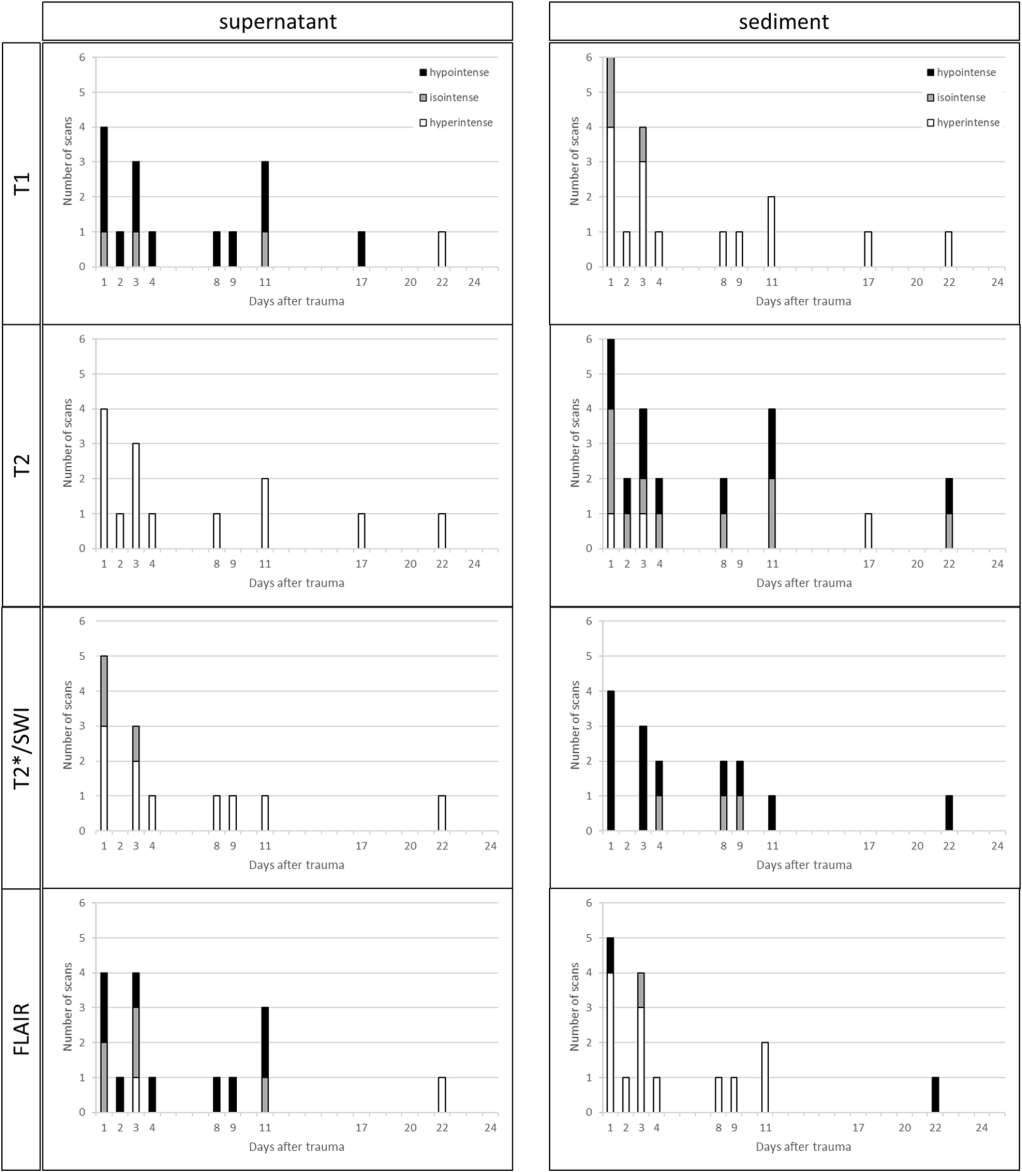
Different signal intensities of supernatant (left column) and sediment (right column) in four different MRI sequences. As a single supernatant or sediment can appear with different signal intensities at different parts, multiple counts are possible. The diagram reveals that, basically, the supernatant resembles the signal intensity of CSF, whereas the sediment rather shows a slight trend toward a time-dependent change in the signal intensity

Different signal intensities of the supernatant and the sediment of the SDCs were found in 15 MRI scans. Figure 4 reveals that, basically, the supernatant resembles the signal intensity of CSF, whereas the sediment rather shows a slight trend toward a time-dependent change in the signal intensity. Evaluation of such MR signal intensities was not available from day 24 due to a lack of sediment and supernatant.

The evaluation of the subdural blood signal intensity at different MR sequences (T1, T2, and FLAIR) showed a main occurrence of a hypo-/isointense signal in T1 between 3 h and 3 d after trauma. Other signal intensities of the blood showed no apparent distribution pattern.

**Fig. 5:**
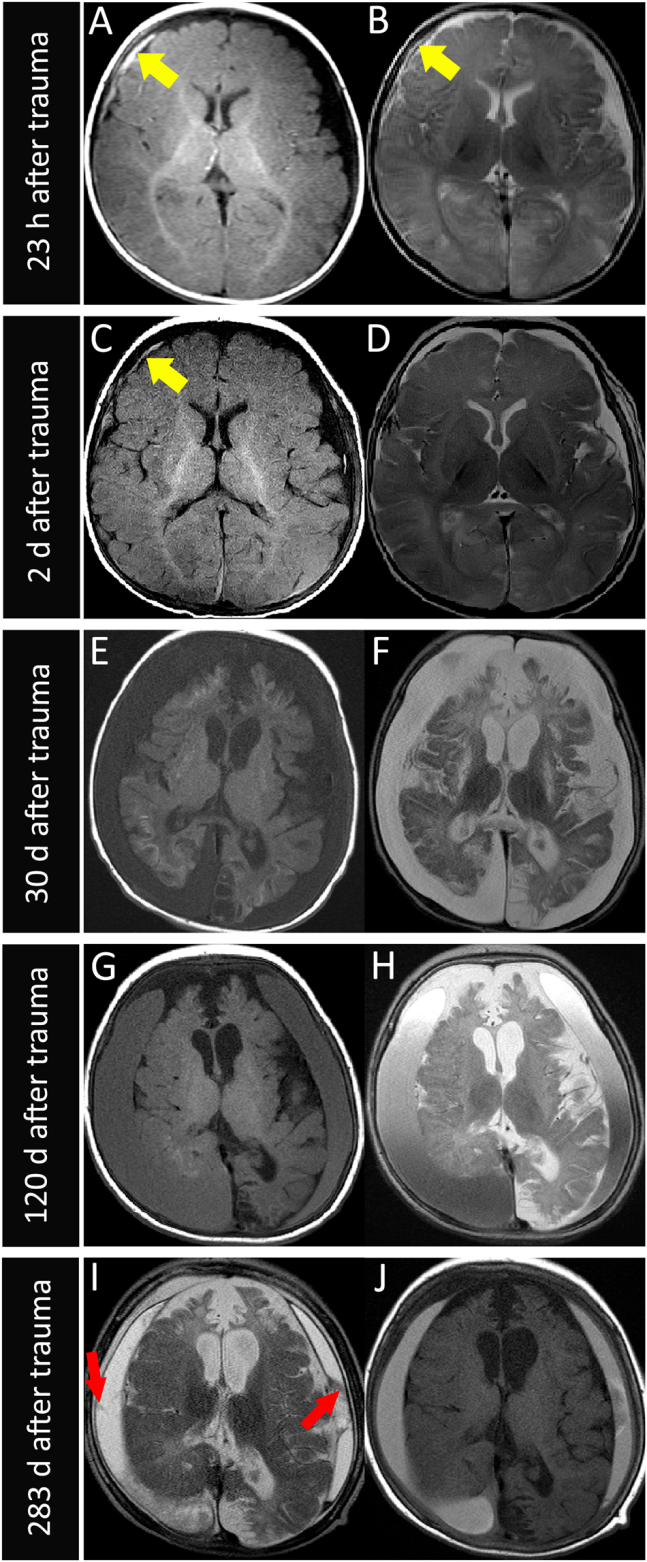
MRI scans of an initial 2-month-old girl with four follow-ups showing a typical transformation of an SDC over time. T1w imaging is shown on the left (A, C, E, G, and I), and T2w imaging is shown on the right (B, D, F, H, and J). Twenty-three hours after trauma (A + B), an SDH (yellow arrows) is detectable on the right convexity. Two days after trauma (C + D), the SDH has noticeably decreased. Thirty days after trauma (E + F), an SDHy with a CSF-like appearance, as well as defects and loss of brain parenchyma have occurred. One hundred twenty days after trauma (G + H), the SDHy has turned into an SDHHy homo with hyperintense (G) and hypointense signal (H) compared with CSF. As the patient did not undergo neurosurgery, the “new” blood component might possibly come from minimal and innocent rebleeding out of fragile new vessels of early neomembranes. Two hundred eighty-three days after trauma (I + J), a cSDH with clearly visible septa and neomembranes (red arrows) has developed

The SDHHy hete (13/26 scans) was found to be the dominant SDC entity in the time between 3 h and 22 d after trauma (Fig. 2). One SDH was observed 23 h after trauma. SDHHy homo was present 19 h, 120 d, and 173 d after trauma. SDHy (5/26 scans) was found at 20 d, 24 d, 30 d, 41 d, and 46 d after trauma. One cSDH was detectable on day 283 after trauma (Fig. 5, I + J). One of the MRI scans (1/26 scans) on day 2 after trauma was not evaluable regarding the SDC entity due to incomplete data. Figure 5 shows a case example with four follow-up MRI scans and a typical SDC transformation over time. Two scans/cases showed no SDC anymore on days 476 and 490 after trauma.

One MRI scan on day 283 showed subdural membrane formation (Fig. 5, I + J).

**Fig. 6:**
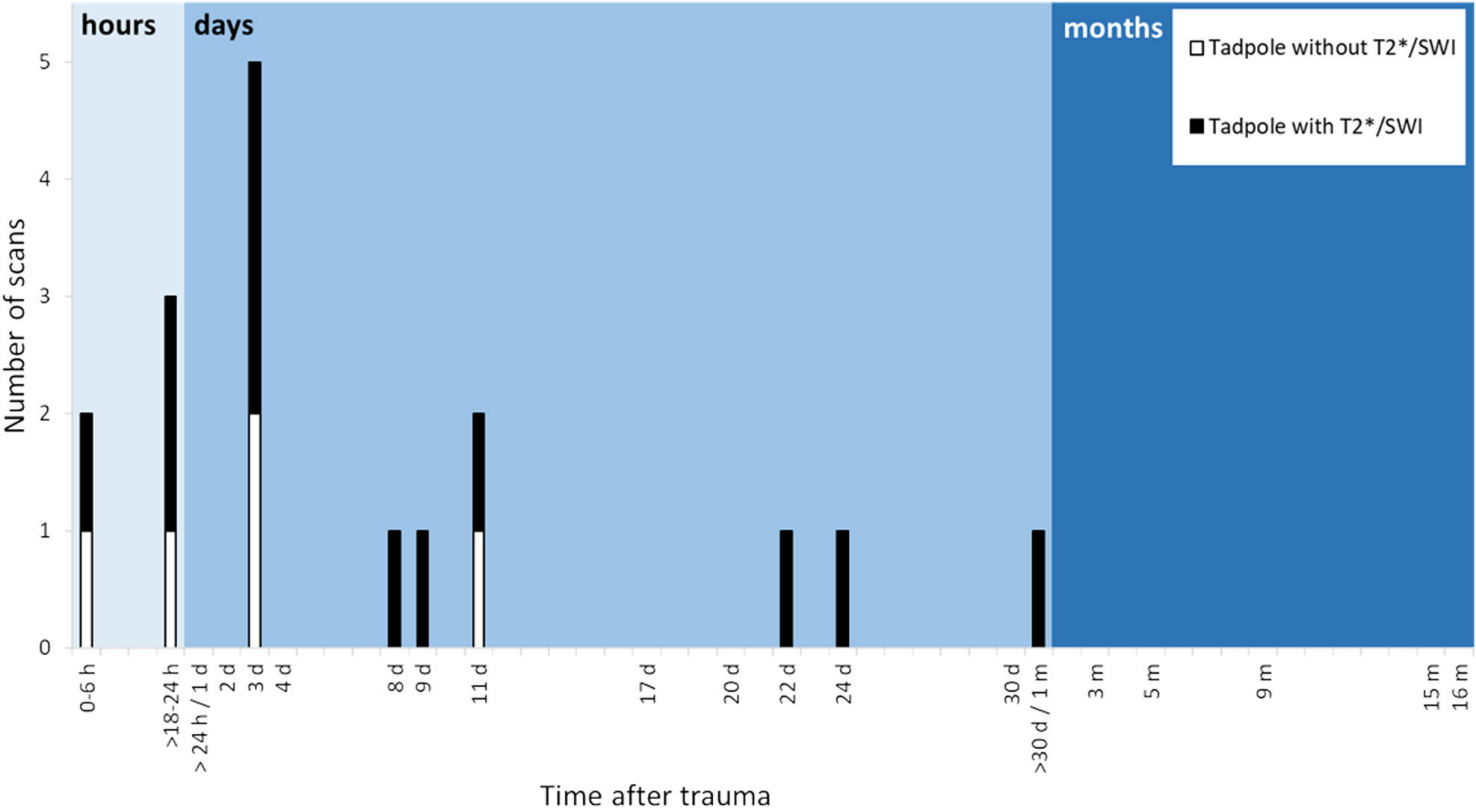
Detection of the tadpole sign in MRI scans at different time points with or without the use of T2*/SWI

The tadpole sign was found between 3 h and 46 d after trauma (17/26 scans; 8 cases). In five of the eight cases, where a tadpole sign was detected, the tadpole sign could not be detected again in later MRI. Figure 6 demonstrates the presence of the tadpole sign over time. The tadpole sign was more often detected when a T2*/SWI sequence (5/26 vs 12/26 scans) was used additionally. One of the MRI scans on day 2 after trauma was not evaluable regarding the presence of the tadpole sign without the use of T2*/SWI due to incomplete data (1/26 scans). Furthermore, seven MRI scans were not evaluable regarding the presence of the tadpole sign with the use of T2*/SWI due to lack of a T2*/SWI sequence (7/26 scans); thereof, two MRI scans on day 2 after trauma (2/26 scans). The tadpole sign was not detectable in MRI scans after day 46, neither with nor without the T2*/SWI sequence.

## Discussion

Estimating the age of injury in pediatric AHT is a challenging task but potentially valuable for the identification of individuals who might have caused the trauma and those who can be excluded. As SDCs represent the most common features diagnosed in neuroimaging of suspicious AHT cases, and as the knowledge on temporal changes of intraparenchymal hemorrhages cannot be simply transferred to extra-axial hemorrhages, several study groups specifically investigated the temporal development of SDCs in CT and MRI. This resulted in a few seminal articles offering time frames for the presence of hyperacute, acute, subacute, or chronic SDCs [10, 15–18] (summarized in Table 1 [9]). However, the available data seem to be incomplete and sometimes even conflicting, mainly attributed to the low number of studies. Thus, the present study aimed at providing more novel reference data on the occurrence and development of SDCs, SDC components, or SDC-associated phenomena, supplementing the existing database and taking a step towards more reliable age diagnostics in the future.

The present study shows that certain subdural findings in pediatric neuroimaging may be helpful for estimating the age of injury in AHT cases. Table 4 presents a comparison of the novel results with previous studies.

**Table 4:** Comparison of neuroimaging studies on the temporal development of SDCs

	Lee et al [15]	Dias et al [16]	Vinchon et al [10]	Tung et al [17]	Bradford et al [18]	Present study
General study characteristics						
No. of cases	428	33	20	47	97**	13
Type of head trauma	AccHT	AHT	AHT (*n* = 12), AccHT (*n* = 8)	AHT (*n* = 9), AccHT (*n* = 38)	AHT	AHT
Age group	n/a (adults)	1–26 mo.	0–12 mo.	0–34 mo.	0–24 mo.	1–5 mo.
Imaging modalities used	CT	CT and MRI	CT and MRI	CT	CT and MRI	CT and MRI
Study design	Retrosp.	Retrosp.	Prosp./retrosp.	Retrosp.	Retrosp.	Retrosp.
Source of the time of injury	n/a (Medical records?)	Medical charts, time of calls made to EMS, and time at which the ambulance received a call or arrived on the scene*	AHT: reports of judicial hearings made public (incl. date of trauma for all cases); AccHT: obstetrical reports, traffic police reports	AHT: n/a; AccHT: medical charts, traffic accident reports	Medical records, ambulance trip sheets, emergency department records	Confessions made by perpetrators (extracted from the complete court records)
Subdural findings in neuroimaging						
First and last detection of hyperdense SDC (or SDC component) in CT	0–12 d p.i.	n/a	0–28 d p.i.	0–9 d after AccHT	0–40 d p.i.	0–9 d p.i.
First detection of CSF-like SDC (or SDC component) in CT or MRI	4 d p.i.	19.75 h p.i.	9 d p.i.	1 d after AccHT	0.3 d p.i.	3 h p.i.
Subdural clot (MRI)	n/a	n/a	0–6 d p.i.	n/a	n/a	0–11 d p.i.
Sedimented blood (MRI)	n/a	n/a	3–20 d p.i.	n/a	n/a	0–22 d p.i.
Tentorial sediment (MRI)	n/a	n/a	4–9 d p.i.	n/a	n/a	0–9 d p.i. 0–22 d p.i.^#^
First detection of the subdural membrane(s)	n/a	n/a	3 d p.i.	n/a	n/a	283 d p.i.
First and last detection of the tadpole sign	n/a	n/a	n/a	n/a	n/a	0–11 d p.i. (0–22 d p.i.)^#^

*AccHT* accidental head trauma, *mo.* months, *n/a* not available, *p.i.* post-injury, *EMS* emergency medical services

^*^ The “time between the alleged injury and the scan” was recorded

^**^ In 97 of 105 cases, there were “SDH” with “identifiable date of injury”

^#^ Using T2*/SWI

The presence of hyperdense SDCs in CT was found up to 9 d post-injury (p.i.), which perfectly agrees with Tung et al [17]. However, all other comparative studies found hyperdense SDCs beyond day 9 as well [10, 15, 16, 18]. The upper maximum is currently described as day 40 [18]. It must be noted that this strikingly late observation could also be a “wrong-positive” result of repeated violence, or could be due to spontaneous and innocent rebleeding phenomena in the context of cSDH formation. Moreover, it has to be considered that the present study only included one CT scan after day 9, so there could be an inclusion bias as well.

The first detection of CSF-like SDCs or SDC components (probably due to CSF influx following arachnoid tears, “hypodense subdurals” in CT), which are primarily part of SDHHys, was found as early as from the 3rd hour p.i. Likewise, two other study groups have already observed this early occurrence of supposedly “chronic” SDCs [16–18], corroborating the recommendation that such SDC components must not be interpreted as a sign of chronicity or old trauma. Nevertheless, this is still often claimed in clinical radiological reports.

The time intervals of the detection of subdural clots, subdural sediment formation, and tentorial sediment found in the present study are nearly in line with the previous data by Vinchon et al [10]. Slight differences might be explained by the low case numbers of both studies. Unsurprisingly, the usage of T2*/SWI sequences increased the time of detectability of blood components on the tentorium.

Subdural membranes were found as late as day 283 in the present study cohort, confirming this feature as a late phenomenon after sustaining AHT. Accordingly, no other comparative study described subdural membranes in their case samples, except one. Vinchon et al [10] mentioned one case with subdural membranes already on day 3 p.i. However, considering the pathogenesis, it seems highly likely that this could have been a case of repeated head trauma with the rare development of acute SDC formations into cSDH. In MRI, the first detectability of subdural neomembranes is classically described after approximately 2–4 weeks [7], whereas in neuropathology they can be macroscopically visible as early as approximately 10 d [19]. Of course, it must be considered that the relatively late detection of subdural membranes in our study cohort might be due to the low number of neuroimaging studies available between week 2 and day 283 p.i. (*n* = 13/34 scans; 38.2%). Thus, further studies with more reliable data are needed.

The tadpole sign was not detected in CT, possibly due to lower sensitivity for bridging vein thromboses. In MRI, the tadpole sign was found between day 0 and day 11 and day 0 to day 46, respectively, depending on the usage of T2*/SWI sequences. These findings support the hypothesis that, as assumed previously [14], the tadpole sign is rather a phenomenon of the early phase after head trauma. However, comparative data are not available yet. Further studies are needed to evaluate the potential of this feature for age diagnostics.

The present study additionally supports previous data by Vinchon et al [10] that, in the case of separation of the SDC into supernatant and sediment, only the sediment may have the potential to be valuable for age-diagnostic aspects.

Inherently, the present study has limitations but also strengths. In a recent review article, Dias and Thamburaj [20] pointed out that there are four major limitations to all studies of neuroimaging in infants with AHT. At first, neuroimaging is usually “performed for clinical indications” leading to “large gaps in time between scans”. This is also the case in the present study and will probably always be so in future studies as long as mere forensic (cross-sectional) neuroimaging, using shorter and uniform time intervals of repeated imaging, appears virtually impossible due to unavoidable requirements, such as minimization of exposure to radiation in CT, or sedation in pediatric MRI.

The second important limitation [20] pertains to the “unknown time of the actual injury”. In contrast to most other studies (Table 4), the present study avoids this by only including confirmed AHT cases with a known date of trauma corroborated by confessions. Moreover, further standardization was achieved by the collection of AHT cases caused by only one trauma mechanism (violent shaking of the child). This approach increases the reliability of the results compared to studies that also included unconfirmed AHT cases, different trauma mechanisms, or unclear dates of trauma. On the other hand, our study approach restricted the number of cases to only 13, despite a multi-center approach with a 10-year study period. The problem of low case numbers is in line with the seminal study by Vinchon et al [10], which was able to consider only 20 infants with corroborated head injury (ten cases due to trauma by “shaking”, four cases of “traffic accident”, four cases of “birth-related trauma”, and two cases of “beating”). However, in contrast to the study by Vinchon et al [10], the present study cohort comprised infants of a small age interval (1–5 months) and a single trauma mechanism, thereby further increasing the comparability of the cases.

As a third important limitation, Dias and Thamburaj [20] listed “repeated episodes of abuse”. Hence, in the present study, two cases with known repeated AHT at different time points were excluded. Thereby, a bias due to superimposition phenomena by age-different subdural blood or previous influx of CSF into the subdural space should at least be minimized. Of course, it cannot be excluded that the confessions in the remaining 13 study cases were incomplete (confession of only one trauma instead of multiple incidents).

“Rebleeding into SDHs” without acute symptoms and complicating SDH appearance was mentioned as a fourth important limitation [20]. In the present study, this problem was observed only rarely. This is probably because rebleeding phenomena mainly result from fragile new vessels of neomembranes in the context of cSDH formation, which is generally rare in infants.

To summarize, the results of the present study suggest that a deeper understanding of the temporal development of SDCs or SDC-associated phenomena may still have the potential to be valuable puzzle pieces for estimating the age of injury in AHT cases. Some subdural features have been identified and corroborated, respectively; e.g., that hypodense SDC components must not be interpreted as a sign of chronicity or old trauma. The requirements for reliable reference studies in this field are complex, e.g., due to the necessity of confirmation of child abuse and the time of injury. Ten years ago, a study group from the Netherlands came in a systematic review article to more pessimistic conclusions as to the forensic potential of the temporal development of SDCs [21]. However, we are convinced that there is a potential, and a great need, for those age estimations in numerous criminal and civil proceedings. Of course, the present work on SDC development can only represent another small step towards more reliable age-diagnostic statements in court. Therefore, further reference data are needed. Considering the scarcity of well-documented cases including confessions, larger data sets will probably require international collaboration of multiple AHT study groups.

## Abbreviations

AHT: Abusive head trauma
cSDH: Chronic subdural hematoma
CSF: Cerebrospinal fluid
p.i.: Post injury
SDC: Subdural collection
SDH: Subdural hematoma
SDHHy hete: Subdural hematohygroma, heterogeneous variant
SDHHy homo: Subdural hematohygroma, homogeneous variant
SDHy: Subdural hygroma
